# Effects of physical exercise on physical and mental health-based on the test of mediation and moderation effect

**DOI:** 10.3389/fpsyg.2025.1614888

**Published:** 2025-07-15

**Authors:** Xiaomei Mo, Wenlong Zhao

**Affiliations:** School of Humanities and Social Sciences, Xi’an Jiaotong University, Xi’an, China

**Keywords:** physical exercise, life satisfaction, physical health, mental health, social class

## Abstract

**Introduction:**

Health is not only a personal issue but also a social issue. China’s 13th Five-Year Plan proposes to “develop sports, promote fitness for all, and enhance people’s physical fitness.” In previous studies, scholars have examined the effects of physical exercise on physical and mental health. However, there is currently a paucity of research exploring the underlying mechanisms through which physical exercise impacts mental and physical health.

**Methods:**

In this study, 9,954 sample data points from the 2018 China Labor Force Dynamics data were selected to verify the relationship between physical exercise, life satisfaction, and physical and mental health using Ordinary Least Squares regression. The Karlson-Holm-Breen method was used to test the mediating effect of life satisfaction in the effect of physical exercise on residents’ physical and mental health, and an interaction term was used to test the moderating effect of social class in the effect of physical exercise on residents’ physical and mental health.

**Results:**

We obtained four conclusions: first, there is a significant positive correlation between physical exercise and residents’ physical and mental health. Second, life satisfaction partially mediated the effects of physical exercise on residents’ physical and mental health, with indirect effects of 13.99 and 25.64%, respectively. Third, social class played a negative moderating role in the effects of physical exercise on residents’ physical health (*b* = −0.014,*p* < 0.001) and mental health (*b* = −0.072, *p* < 0.05), i.e., the effects of physical exercise on residents’ physical and mental health gradually decreased as social class increased. Four, the impact of physical exercise is greater on urban residents than on rural residents, and it has no significant effect on the mental health of rural residents.

**Conclusion:**

This study reveals a new mechanism by which physical exercise affects residents’ physical and mental health through life satisfaction, and further explores the moderating role of social class, with conclusions similar to the resource substitution theory. In theory, it expands the application of resource substitution theory in the field of health research. In practice, it provides a basis for the government to continue to develop sports and promote health for all so as to better promote people’s health and equality. This study innovatively introduces a dual-path model integrating life satisfaction as a mediator and social class as a moderator to reveal the mechanisms by which physical exercise affects health. By examining urban–rural differences, it further highlights structural inequalities in health promotion.

## Introduction

With the development of socioeconomic levels and improvement in people’s living standards, there is increasing attention and emphasis on physical and mental health. Concurrently, health research has become an important topic of concern among scholars. Many studies have demonstrated the beneficial effects of regular physical activities and exercise on physical health (Warburton and Bredin, 2018; Warburton and Bredin, 2017; Warburton and Bredin, 2016; Warburton et al., 2006a; Warburton et al., 2006b). Virtually everyone can benefit from becoming more physically active (Warburton and Bredin, 2017). Physical exercise is part of people’s social life, and those who regularly participate in physical exercise and competition are better able to recognize and deal with the relationship between people, people and teams, as well as between competition and cooperation (Tao et al., 2022). Different researchers focus on social group differences in physical exercise and the intrinsic relationship between duration and intensity of physical exercise and health. Some studies suggest that Physical exercise is associated with reduced risk of cardiovascular diseases (CVD), certain cancers, and improvement in mental health and quality of life (Lawlor and Hopker, 2001). Literature indicates a relationship between physical fitness and the intensity of exercise, with greater benefits to health from more Physical exercise. Experimental research findings also demonstrate that moderate Physical exercise can be beneficial for the health of high-risk adolescents, such as those with obesity (Janssen and AG, 2010). Other scholars have found through survey research that individuals who maintain physical exercise from adolescence to young adulthood have a significantly lower risk of developing cardiovascular diseases compared to those who do not exercise. Additionally, they exhibit better mental health conditions (Rangul et al., 2012; Hou et al., 2024).

Life satisfaction, which is a person’s evaluation of his or her life based on the factors he or she considers most relevant (Diener et al., 1985), is a promising health asset (Kim et al., 2021). It is not only influenced by genetics, social structure, and living environment (Barger et al., 2009; Diener et al., 2018), but can also be intervened through a number of measures, such as physical exercise (Wiese et al., 2018). Studies have shown that physical exercise can promotes life satisfaction. Some studies have discussed the impact of physical exercise on life satisfaction. Whether in adolescents (Slapšinskaitė et al., 2020; Reigal et al., 2014; Valois et al., 2004), middle-aged individuals (An et al., 2020), or older adults (An et al., 2020; Inal et al., 2007), physical exercise can enhance individual life satisfaction. Moreover, there is heterogeneity in the effects of exercise intensity, frequency, and type on life satisfaction (Pedišić et al., 2015). In addition, higher life satisfaction is associated with better health outcomes (Kim et al., 2021). Life satisfaction is not only related to subjective health assessments (Ziolkowski et al., 2015), but there is a significant negative correlation with depressive symptoms (Moksnes et al., 2016). Current research primarily explores the effects of different types and intensities of physical exercise on the health or life satisfaction of various demographic groups such as different age groups, genders, and social classes. However, life satisfaction is different for those who regularly participate in physical exercise, which positively affects individuals’ self-rated health as well as their psychological well-being (Kim et al., 2021). Thus, whether physical exercise affects physical and mental health through life satisfaction is a topic worth exploring.

The relationship between social class and physical and mental health has been an important element of health inequality research. Social causation theory suggesting that an individual’s level of health can be limited by social structural factors. An individual’s position in the social structure determines their level of health, with lower socio-economic status correlating with worse health (Dahl, 1996). Whereas physical exercise can improve an individual’s physical and mental health, there has been less discussion about whether its facilitating effect is heterogeneous for individuals from different social classes. In the field of health research, some scholars have found the law of diminishing marginal output (Grossman, 2017), that is, the physical and mental health returns to physical exercise are smaller for groups of higher social class than for groups of lower social class. Therefore, we believe that individuals from different social classes may experience different levels of physical and mental health changes when participating in physical exercise, and that the specific differences need to be further investigated.

Previous studies have mainly explored the relationship between the two separately for physical exercise, life satisfaction, and physical and mental health, and rarely explored the relationship among all three. This study selects data from CLDS 2018 and employs methods such as multiple linear regression and KHB tests. Building upon existing theories and research, this study explores the mediating effect of life satisfaction in the impact of physical exercise on both physical and mental health. To understand the differential effects of physical exercise on the health of different social classes, social class is incorporated as a moderating variable to examine its regulatory role and better reveal underlying patterns. Additionally, given China’s urban–rural dual structure, significant disparities exist between cities and rural areas in terms of economic development, infrastructure, and public services. Persistent differences in physical exercise participation between urban and rural residents also remain (Lu and Miao, 2024). Therefore, it is necessary to further examine whether there are urban–rural differences in the health effects of physical exercise.

## Literature review and hypotheses

### Physical exercise and physical and mental health

Currently, the consensus regarding the impact of physical exercise on physical and mental health is quite consistent. Generally speaking, both regular physical exercise and sports activities are beneficial to health (Warburton and Bredin, 2018; Warburton and Bredin, 2017; Warburton and Bredin, 2016; Warburton et al., 2006a; Warburton et al., 2006b). Engaging in regular physical exercise or sports activities is considered beneficial for preventing more than 25 chronic diseases, serving as primary or secondary prevention measures (Warburton and Bredin, 2018; Warburton and Bredin, 2017; Warburton and Bredin, 2016; Warburton et al., 2006a; Warburton et al., 2006b; Warburton et al., 2007; Gledhill et al., 2016). The intensity of physical exercise also significantly influences physical health; higher levels of physical exercise can reduce the risk of premature death and chronic diseases by 20–30% (Warburton and Bredin, 2016; Gledhill et al., 2016; Paterson and Warburton, 2010). In a recent systematic review of the literature, researchers uncovered compelling data (from millions of participants) (2) showing that the amount of regular physical exercise is associated with reduced risks of various diseases (such as cardiovascular diseases, all-cause mortality). In the majority of studies, a non-linear relationship exists, where individuals who do not typically engage in physical exercise experience the greatest health benefits from starting to exercise. Importantly, studies have demonstrated that exercise can bring significant health benefits.

Furthermore, the benefits of physical exercise on mental health have also been demonstrated by many scholars. Literature shows that individuals who maintain physical exercise from adolescence to young adulthood not only experience significantly reduced risks of cardiovascular diseases but also exhibit better mental health conditions compared to those who are inactive (Warburton et al., 2007). Studies have also demonstrated that regular physical exercise not only improves mental health (by reducing stress, anxiety, and depression) (Warburton et al., 2001a; Warburton et al., 2001b; Dunn et al., 2001) but also prevents the development of psychological disorders (Lubans et al., 2016; Biddle et al., 2019; Liming and Jing, 2019). Research on different types of sports, diverse populations, and various age groups has yielded similar conclusions (Biddle et al., 2019; Heo et al., 2013; Zayed et al., 2018; Beauchamp et al., 2018). The majority of studies indicate that moderate exercise can benefit both physical and mental health (Niu, 2024; Liu et al., 2024; Feng et al., 2024).

However, in real life, researchers have different perspectives on how to define “moderate physical exercise.” Research has found that while physical exercise promotes individual physical and mental health, excessive exercise can have adverse effects. Individuals who exercise more than 23 times per month or for more than 90 min per session tend to experience worse mental health (Chekroud et al., 2018). Further studies reveal that, compared to exercise intensity and duration, exercise frequency has a greater impact on mental health, and this effect varies by gender (Grasdalsmoen et al., 2020). Additionally, due to differences in economic status, living environments, and lifestyle attitudes between urban and rural residents in China, there are disparities in their participation in physical exercise (Lu and Miao, 2024) and their levels of physical and mental health (Liang and Jia, 2022). Research has shown significant differences in exercise levels between urban and rural elderly populations. The proportion of elderly people in rural areas who do not engage in physical exercise is relatively high, while the proportion of elderly people in urban areas who engage in moderate to intense levels of exercise is higher than that of rural elderly individuals. Additionally, the impact of different levels of physical exercise on mental health also shows urban–rural differences (Fangfang et al., 2021). Some scholars have found through urban–rural comparisons that the effects of physical exercise on the physical health and mental health of rural residents are smaller than those of urban residents (Liang and Jia, 2022). However, other studies have found no significant urban–rural differences in the effects of physical exercise on mental health (Liming and Jing, 2019). Therefore, it is necessary to further examine the urban–rural differences in the impact of physical exercise on physical and mental health.

Based on the above arguments, the following hypothesis is proposed for this study: Physical exercise has a positive impact on physical and mental health. Specifically, this can be categorized into:

*H1-1*: Physical exercise has a positive impact on physical health.

*H1-2*: Physical exercise has a positive impact on mental health.

*H1-3*: The impact of physical exercise on residents’ physical and mental health exhibits urban–rural differences, with the health-promoting effects of physical exercise being stronger for urban residents compared to rural residents.

### Effects of physical exercise on physical and mental health: the mediating role of life satisfaction

Currently, there is no unified definition of life satisfaction. Activity theory suggests that life satisfaction is influenced by the frequency of participation in specific activities and the degree of closeness associated with these activities. Higher frequency and greater intimacy lead to higher life satisfaction (Havighurst, 1961). Although activity theory was initially developed to explain successful aging, its premise regarding life satisfaction has been confirmed in general adult populations (Rodríguez et al., 2008). Compared to individuals who do not engage in physical exercise, those who do report higher life satisfaction (Reigal et al., 2014; Yazicioglu et al., 2012). A review of past research reveals that regular physical exercise enhances life satisfaction across all age groups, including older adults (Clark et al., 1999; Elavsky et al., 2005), adults (Eime et al., 2010; Schnohr et al., 2005), youth (Warburton et al., 2007; Joseph et al., 2014; Vaez and Laflamme, 2003; Maher et al., 2013), and children and adolescents (Valois et al., 2004; Proctor et al., 2009; Zullig and White, 2011; Rangul et al., 2011). Certain types of physical activities such as stretching (Valois et al., 2004), jogging (Schnohr et al., 2005), strength training (Valois et al., 2004; Rodríguez et al., 2008), and walking (Clark et al., 1999; Morgan and Bath, 1998) are positively correlated with life satisfaction. However, some scholars, through a separate survey of rural Australian women’s physical exercise in sports clubs, gyms, and walking, found that the significant association between physical exercise and life satisfaction is only related to the type of exercise, not the level of exercise (Eime et al., 2014). In summary, the analysis shows that although there are differences in the effects of different types of physical exercise, frequency of exercise, and duration of exercise on life satisfaction, overall there is an effect of physical exercise on life satisfaction (Ha and Zhang, 2024), and as a result, the hypothesis is formulated:

*H2-1*: Physical exercise has a positive impact on life satisfaction.

Currently, there is limited research on the impact of physical exercise on life satisfaction that addresses urban–rural differences. However, research on life satisfaction reveals significant urban–rural disparities across different groups, including the elderly (Li and Liu, 2015; Yu, 2017), university students (Zhang and Yao, 2011), and primary/secondary school students (Meng, 2014). Even after controlling for major factors influencing life satisfaction, these disparities persist, with urban residents reporting higher life satisfaction than their rural counterparts (Li and Liu, 2015; Yu, 2017). From this, we can infer that the impact of physical exercise on life satisfaction also varies between urban and rural areas. Based on this, the hypothesis can be proposed:

*H2-2*: The impact of physical exercise on life satisfaction exhibits urban–rural differences.

Regarding the impact of life satisfaction on physical and mental health, some scholars have explored this topic, but the research conclusions are not consistent. Studies indicate that higher life satisfaction among older adults is associated with higher self-rated health scores, better mental health, and lower levels of depression, despair, and loneliness (Kim et al., 2021; Ziolkowski et al., 2015). Additionally, higher life satisfaction can reduce the risk of chronic diseases (Boehm et al., 2011; Feller et al., 2013) and decrease mortality rates (Martín-María et al., 2017). Poor physical and mental health states over the past 30 days are significantly correlated with lower life satisfaction (Valois et al., 2004), and women with lower life satisfaction have a higher probability of developing cancer and stroke (Feller et al., 2013). Other scholars have reached different conclusions. Through studies on older adults, some researchers found that life satisfaction does not have a significant impact on physical health (Gana et al., 2013). In summary, although the conclusion of the impact of life satisfaction on physical and mental health is not consistent, most scholars believe that live satisfaction can have a positive impact on physical and mental health. Based on this, the hypothesis is proposed:

*H3-1*: Life satisfaction has a positive impact on physical health.

*H3-2*: Life satisfaction has a positive impact on mental health.

Based on the previous overview of life satisfaction of urban and rural residents, it is known that there are significant differences in life satisfaction between urban and rural residents, so we can infer that there will be differences in the impact of different life satisfaction on physical and mental health. As a result, the hypothesis is proposed:

*H3-3*: The impact of life satisfaction on residents’ physical and mental health exhibits urban–rural differences.

Research indicates that physical exercise can enhance adolescents’ subjective well-being through the mediating effect of life satisfaction, thereby contributing to their physical and mental health (Tao et al., 2022). Moreover, physical exercise can improve the anti-depressive ability of the elderly by enhancing their life satisfaction (Yang et al., 2025). From the literature review above, it is evident that physical exercise has a positive impact on both physical health and mental health, as well as life satisfaction. Furthermore, life satisfaction can influence physical and mental health. Therefore, We can propose the following hypotheses: physical exercise has a positive impact on physical and mental health through increasing life satisfaction, as follows:

*H4-1*: Physical exercise has a positive impact on physical health through increasing life satisfaction.

*H4-2*: Physical exercise has a positive impact on mental health through increasing life satisfaction.

Based on the previous analysis, it can be seen that there are urban–rural differences not only in residents’ physical exercise, but also in residents’ life satisfaction, from which it can be inferred that there are urban–rural differences in the mediating effect as well; therefore, it is further hypothesized:

*H4-3*: The mediating effect of life satisfaction on the relationship between physical exercise and physical/mental health exhibits urban–rural differences.

### Effects of physical exercise on physical and mental health: the moderating role of social class

There is already a substantial body of research on the relationship between social class and health inequality. Social class is a relative social ranking formed by differences in economic capital, social capital, and cultural capital among organization members (Gray et al., 2013). Social class not only implies differences in the distribution of wealth, resources, and status but also suggests unequal distribution of discrimination, pain, and illness (Guo, 2016). Existing research has shown that social class, especially subjective social class, can predict health outcomes (Operario and Adler, 2004), with lower subjective social class being associated with poorer self-rated health (Goodman et al., 2007). Individuals with higher social status can access more social, economic, and psychological resources and tend to have healthier lifestyles (Ross and Wu, 1995), as well as a preference for acquiring and maintaining healthy lifestyles (Wang, 2012). At the same time, they also have access to fitness equipment and higher-quality healthcare services, which can reduce health risks (Guo, 2016). Lower social class groups have increased health risks due to more unhealthy lifestyles (Wang, 2012). In terms of psychological factors, the impact of social class on health includes not only psychological stress and depression caused by social resources and interactions but also further resulting in physical ailments and self-harm (Martín-María et al., 2017).

It is almost common knowledge that physical exercise can promote physical health. However, little attention has been paid to whether there are differences in the effects of physical exercise on the physical and mental health of people from different social classes. In this study, we draw on the ‘resource substitution’ and ‘resource enhancement’ theories proposed in the field of medical sociology in relation to the study of the relationship between education and health (Ross and Mirowsky, 2006). The resource substitution theory suggests that groups with fewer other types of resources are more dependent on scarce educational resources, and therefore gain more benefits from education. The resource enhancement theory, on the other hand, argues that the positive impact of education on health creates a ‘Matthew effect,’ meaning individuals with more other social resources gain more from educational achievements, leading to the phenomenon where ‘the strong getting stronger and the weak getting weaker.’ Whether it is ‘resource substitution’ or ‘resource enhancement,’ both theories assert that the same level of education has different effects on men and women. Drawing on these two theories, we can infer that, because individuals from different social classes have unequal access to physical exercise resources, the health benefits of physical exercise vary across different social strata. Additionally, in health research, some scholars have identified the law of diminishing marginal returns, which suggests that when it comes to reducing health risks, the same investment in resources has a smaller impact on groups with higher health stocks (higher socioeconomic status) compared to those with lower health stocks (lower socioeconomic status). This is similar to the resource substitution theory.

Some studies have found that the health effects of physical exercise vary among residents of different social classes, with a greater anti-depressive effect on residents of lower social classes, thereby promoting better psychological health, while having no significant effect on the psychological health of residents of higher social classes (Liming and Jing, 2019; Lorant et al., 2003). At the same time, square dancing had a significantly higher impact on self-rated health and physical health and mental health in older adults of lower social class than in older adults of higher social class (Yuan, 2019). These research conclusions align with the “resource substitution” theory and the law of diminishing marginal returns. However, scholars have reached different conclusions through surveys and studies in China, suggesting that as social class increases, the anti-depressive effect of physical exercise becomes stronger, meaning that physical exercise has a greater anti-depressive effect on residents of higher social classes (Wang et al., 2022), a conclusion also supported by American scholars (Wann et al., 2011). This is consistent with the “resource enhancement” theory.

Although current research has not yet reached a unified conclusion regarding the moderating role of social class in the impact of physical exercise on residents’ mental health—whether “resource substitution” or “resource enhancement” is more explanatory still requires further exploration—the persistent urban–rural disparities also call for a comparative study of the moderating role of social class in both urban and rural contexts. Therefore, we can propose the following hypotheses:

*H5-1*: Social class moderates the impact of physical exercise on residents’ physical health.

*H5-2*: Social class moderates the impact of physical exercise on residents’ mental health.

*H5-3*: The moderating effect of social class on the relationship between physical exercise and residents’ mental health exhibits urban–rural differences.

## Materials and methods

### Data sources

The data used in this study is derived from the China Labor-force Dynamics Survey (CLDS), conducted by the Social Survey Research Center of Sun Yat-sen University. The CLDS focuses on the current status and dynamic changes of China’s labor force, encompassing areas such as education, employment, health, and migration. The survey covers 29 provinces and municipalities across China, excluding Hong Kong, Macau, Taiwan, Tibet, and Hainan, and targets all labor force members within sampled households. It adopts a multistage, stratified sampling method with probability proportional to size. For panel tracking, the survey employs a rotating sample approach, following up with households in selected communities every 2 years to adapt to environmental changes and maintain sample representativeness. The data is considered high quality and has supported the publication of numerous academic studies both domestically and internationally. The most recent round of data collection was conducted in 2018. Subsequently, based on the needs of the study, samples with missing key variables—primarily the individual income variable (with 4,666 missing cases)—were excluded, resulting in a final valid sample of 9,954.

### Variable selection

#### Dependent variables

The dependent variables in this study are physical health and mental health. The 5-point self-rated health scale proposed by the World Health Organization (WHO) has become a standard tool for health surveys. This study also selects this scale, asking the question, “How do you rate your current health?” The answers are assigned values of 1–5 on a Likert scale. During data analysis, reverse coding is applied, where higher values indicate higher levels of physical health. The measurement of mental health selects the Center for Epidemiologic Studies Depression scale 20 (CES-D20) to assess respondents’ levels of depression. The scale consists of 20 items, asking respondents to report the frequency of 20 symptoms experienced in the past week. The options include: not at all/almost not at all (less than 1 day), occasionally (1–2 days), frequently (3–4 days), or almost always (5–7 days). These responses are scored as 0–3 points, respectively. The sum of these scores is used as a proxy variable for mental health. Reverse coding is also applied during data analysis, where higher scores indicate higher levels of psychological health. The final value of this variable ranges from 0 to 60.

#### Independent variable

The core independent variable in this study is physical exercise, and we measure physical exercise time. The questionnaire first asks ‘whether residents engaged in regular physical exercise in the past month’. For those who reported regular physical exercise, the questionnaire further inquires about exercise frequency (the average number of times per week they exercised) and exercise duration (the average duration of exercise per day). Exercise frequency and duration are only applicable to respondents who have engaged in regular physical exercise in the past month, and this subgroup accounts for 32.50% of the total sample. To retain as many samples as possible, respondents who did not engage in physical exercise in the past month were assigned a frequency and duration of 0. The calculation of average daily exercise time draws on the approach used in similar studies (Hu and Yu, 2019): First, Extreme values exceeding 360 min (6 h) per exercise session were removed. Then, the average daily exercise time was calculated as follows: Average daily exercise time = (Number of exercise days per week × Duration of exercise per day)/7. To make the variable more closely conform to a normal distribution, this study adds 1 to the average daily exercise time and then takes the logarithm of the result, thereby constructing a continuous variable that follows a normal distribution. The final value of this variable ranges from 0 to 7.16.

#### Mediating variable

The mediating variable in this study is life satisfaction. In the CLDS questionnaire were asked residents: “Overall, how satisfied are you with your life?” Responses were assigned values using a Likert scale, where satisfaction levels were scored from low to high, ranging from 1 to 5 points. Higher scores indicate higher levels of life satisfaction.

#### Moderating variable

The moderating variable in this study is social class, specifically residents’ subjective perception of their social class. Although subjective class perception may differ from objective class measures, it allows individuals to compare their income, wealth, and status within their specific social context, thereby gaining a sense of their relative social position. Objective social class classifications, on the other hand, often fail to capture these nuanced self-assessments. Residents were asked in the CLDS questionnaire: in our society, if a score of 10 represents the top class and a score of 1 represents the bottom class, in what class do you think you are? Answers ranged from 1 to 10, with higher scores indicating higher social class.

#### Control variables

Previous studies have shown that individual’s physical and mental health is influenced by factors such as gender, age, marital status, household registration (hukou), education level, income, social class, and smoking status. This study only examines individuals aged 70 and below. Factors affecting health among individuals aged over 70 are more varied, and respondents are likely to be the healthier ones who were selected. Therefore, the health mechanisms among individuals aged over 70 may differ from other age groups. Therefore, the above variables will be treated as control variables in the analysis. To observe whether age has a nonlinear effect, age squared is included in the model. Family economic satisfaction affects individuals’ life satisfaction and is also included as a control variable in the model.

Descriptions and descriptive statistics of the variables mentioned in the study are presented in Table 1.

**Table 1:** Results of descriptive statistical analysis of relevant variables.

Variable	M (SD/Per)		t or χ2	Variable value
	Urban (2906)	Rural (7048)		
Dependent variable				
Physical health	3.86(0.84)	3.57(0.99)	13.97***	Min = 1, Max = 5
Mental health	53.07(8.67)	52.16(9.17)	4.55***	Min = 0, Max = 60
Independent variable				
Physical exercise	1.50(1.70)	0.75(1.43)	22.54***	Min = 0, Max = 7.16
Mediator variable				
Life satisfaction	3.79(0.87)	3.68(0.89)	5.80***	Min = 1, Max = 5
Controlled variable				
Gender	0.48(0.50)	0.46(0.50)	4.87*	Male = 0, Female = 1
Age	41.97(11.14)	48.80(11.97)	26.39***	Min = 18, Max = 70
Marital status	0.83(0.37)	0.92(0.24)	159.68***	Unmarried = 0, Married = 1
Household registration	0.65(0.47)	0.067(0.25)	(3.9e+03)***	Rural = 0, Urban = 1
Years of education	11.78(3.88)	7.50(3.81)	50.77***	Min = 0, Max = 19
Logarithm of income	10.36(2.11)	9.18(2.56)	22.05***	Min = 1, Max = 14.91
Social class	4.72(1.72)	4.35(1.73)	9.79***	Min = 1, Max = 10
Smoking status	0.25(0.44)	0.34(0.47)	61.96***	Non-smoker = 0, Smoker = 1
Family economic satisfaction	3.34(0.99)	3.18(1.02)	7.42***	Min = 1, Max = 5

### Data analysis

As the variables in this study primarily pertain to health and life satisfaction, a correlation analysis of the main variables was first conducted to verify the relationships among them. Based on a substantial body of literature published in authoritative journals, physical health and mental health are treated as continuous variables, and the ordinary least squares (OLS) regression method is deemed appropriate. Accordingly, this study employs OLS multiple linear regression for the analysis. Prior to regression, a multicollinearity test was conducted on all independent variables. The results indicate that the variance inflation factor (VIF) for each independent variable is below the commonly accepted threshold of 10, and the average VIF is below 2, suggesting that multicollinearity is not a concern in this model.

Firstly, two sets of general linear regressions are established to separately examine the effects of physical exercise and life satisfaction on physical health and mental health. Secondly, the study examines the mediating effect of life satisfaction on the impact of physical exercise on physical and mental health. Mediating effect tests typically include the Sobel test (Sobel, 1982) and the stepwise method (Baron and Kenny, 1986); both methods assume that the product term variables formed by these methods follow a normal distribution, which results in relatively lower statistical power for testing the coefficients. In smaller samples, the bias-corrected bootstrap method is often more effective in reducing errors than other methods (Hayes and Scharkow, 2013). This study explores the impact of physical exercise on physical and mental health through a mediator variable, and uses KHB method to test the effect and size of the mediation (Kohler et al., 2011); the method can be any of Regress, Logit, Ologit, Probit, Oprobit, Cloglog, Slogit, Scobit, Rologit, Clogit, Mlogit, Xtlogit, or Xtprobit, and it can be extended to other models.

Finally, interaction terms are employed to test the moderating effect of social class on the relationship between physical exercise and physical and mental health.

**Figure 1:**
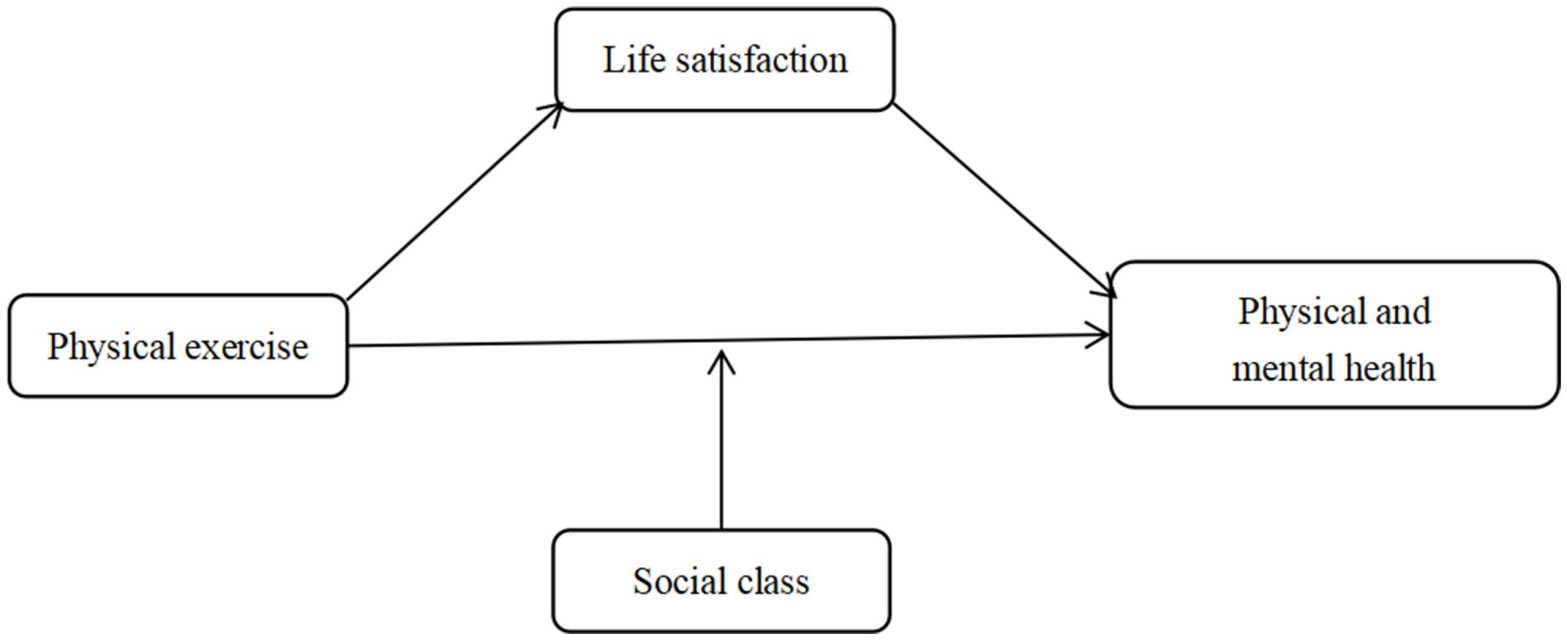
Theoretical model of the effects of physical exercise and life satisfaction on physical and mental health.

The theoretical model of this paper is shown in Figure 1.

## Results

By conducting correlation analyses between physical exercise and physical health, as well as between life satisfaction and mental health, it was found that both physical and mental health are significantly positively correlated with physical exercise and life satisfaction. Additionally, physical exercise and life satisfaction also show a significant positive correlation. The results are presented in Table 2.

**Table 2:** Correlation analysis of variables.

Physical health model				Mental health model			
Variable	Physical health	Life satisfaction	Physical exercise	Variable	Mental health	Life satisfaction	Physical exercise
Physical health	1.0000			Mental health	1.0000		
Life satisfaction	0.2359*	1.0000		Life satisfaction	0.2719*	1.0000	
Physical exercise	0.1051*	0.1144*	1.0000	Physical exercise	0.0773*	0.1144*	1.0000

*** Indicates *p* < 0.001, ** indicates *p* < 0.01, * indicates *p* < 0.05.

### Effects of physical exercise on physical and mental health-promoting

To validate hypothesis 1, we examined the effects of physical exercise on residents’ physical and mental health. The regression analysis results of physical exercise on physical and mental health are shown in Table 3. Overall, physical exercise exerts a significant positive impact on both residents’ physical and mental health. As shown in Full sample Model of Table 3, the longer the average daily physical exercise time, the higher residents’ self-rated health score and better physical health condition (*b* = 0.025, *p* < 0.001). As shown in Full sample Model of Table 3, the longer the average daily physical exercise time, the better residents’ mental health condition (*b* = 0.216, *p* < 0.001). This is because participating in physical exercise is beneficial for resisting and releasing negative emotions, helping to alleviate depressive symptoms. Comparing the effects of physical exercise on physical and mental health, it is found that the impact of physical exercise on physical health (coefficient of 0.025) is much smaller than its impact on mental health (coefficient of 0.216), This is consistent with the conclusion of Liang and Jia (2022), confirming hypotheses H1-1 and H1-2.

**Table 3:** The effect of physical exercise on physical and mental health.

Variable	Full sample model		Urban model		Rural model	
	Physical health	Mental health	Physical health	Mental health	Physical health	Mental health
Gender (female = 1)	−0.117***	−1.312***	−0.077**	−0.706	−0.137***	−1.545***
Age	−0.017***	−0.027**	−0.014	−0.006	−0.019***	−0.034**
Marital status (Married = 1)	0.106**	1.030**	0.031	0.958*	0.150***	0.891*
Household registration (Urban = 1)	−0.087**	−0.015	−0.063*	0.593	0.108*	−0.537
Years of education	0.012***	0.132***	0.009	0.035	0.013***	0.181***
Logarithm of income	0.028***	0.113**	0.013	−0.004	0.031***	0.139**
Social class	0.054***	0.527***	0.052***	0.388***	0.056***	0.606***
Smoking status (smoker = 1)	−0.001	0.310	−0.063	0.99*	0.013	−0.066
Family economic satisfaction	0.210***	1.870 ***	0.172***	1.355***	0.224***	2.094***
District (rural = 1)	−0.077*	0.496				
Physical exercise	0.025***	0.216***	0.029**	0.343***	0.023**	0.128+
Cons	3.207***	42.126***	3.437***	44.978***	3.060***	41.782***
N	9,954	9,954	2,906	2,906	7,048	7,048
Adjusted *R^2^*	0.167	0.093	0.129	0.053	0.160	0.112

*** Indicates *p* < 0.001, ** indicates *p* < 0.01, * indicates *p* < 0.05.

The urban and rural models illustrate the influence of residential location on the health-promoting effects of physical exercise. Overall, the impact of physical exercise on both physical and mental health is weaker among rural residents compared to their urban counterparts. In the rural model, the absolute values of the regression coefficients for physical exercise on self-rated physical and mental health are smaller than those observed in the urban model, and the effect on mental health is not statistically significant, thereby confirming Hypothesis H1-3. A comparison of the two models also indicates that variables such as gender, age, education level, economic status, and social class exert a greater influence on the physical and mental health of rural residents than on those living in urban areas. Additionally, residents’ physical and mental health are significantly affected by demographic characteristics, as shown in the full sample model in Table 3. Women report significantly lower levels of self-rated physical and mental health compared to men. As age increases, self-rated physical and mental health scores decline. Married individuals report better self-rated health and mental well-being than those who are unmarried. Higher levels of education are positively associated with better physical and mental health. Similarly, individuals with better socioeconomic status, higher levels of household economic satisfaction, and higher perceived social class report improved self-rated physical and mental health. While residents with urban household registration tend to report lower self-rated health scores compared to those with rural registration, individuals actually living in urban areas report higher self-rated health scores than those residing in rural areas.

### Effects of physical exercise on residents’ physical and mental health: the mediating effect of life satisfaction

From Table 4, Full sample model, it can be seen that physical exercise has a significant positive effect on residents’ life satisfaction. That is, the longer the duration of physical exercise, the higher the life satisfaction of residents. However, the urban–rural difference is not significant. According to the urban and rural models, there is no significant urban–rural difference in the impact of physical exercise on life satisfaction (the SUR test also yielded the same result). Thus hypothesis 2–1 is validated, but hypothesis 2–2 is not supported.

**Table 4:** The differentiation effect of physical exercise on life satisfaction between rural and urban residents.

Variable	Full sample model	Urban model	Rural model
	Life satisfaction	Life satisfaction	Life satisfaction
Physical exercise	0.032***	0.033***	0.033***
District (rural = 1)	0.024		
Control variable	Controlled	Controlled	Controlled
Cons	1.654***	1.806***	1.636***
*N*	9,954	2,906	7,048
Adjusted *R^2^*	0.347	0.374	0.334

*** Indicates *p* < 0.001, ** indicates *p* < 0.01, * indicates *p* < 0.05.

From Table 5, Full sample model, it is evident that life satisfaction has a significant positive effect on residents’ physical and mental health. The higher the life satisfaction, the higher the scores for residents’ physical and mental health. For each unit increase in life satisfaction, residents’ physical and mental health scores increase significantly by 0.113 and 1.743 points. Comparing the urban–rural models, it can be seen that, compared to urban residents, the impact of life satisfaction on both the physical and mental health of rural residents is smaller. In the rural model, the absolute values of the regression coefficients for life satisfaction on self-reported health and mental health are both smaller than those in the urban model.(the SUR test also yielded the same result). Respectively, thus validating hypotheses H3-1, H3-2, and H3-3 are validated.

**Table 5:** The differentiation effect of life satisfaction on physical and mental health between rural and urban residents.

Variable	Full sample model		Urban model		Rural model	
	Physical health	Mental health	Physical health	Mental health	Physical health	Mental health
Life satisfaction	0.113***	1.743***	0.127***	2.018***	0.108***	1.634***
District (rural = 1)	−0.085**	0.415				
Control variable	Controlled	Controlled	Controlled	Controlled	Controlled	Controlled
Cons	3.014***	39.205***	3.184***	41.088***	2.878***	39.091***
N	9,954	9,954	2,906	2,906	7,048	7,048
Adjusted *R^2^*	0.173	0.111	0.136	0.075	0.165	0.128

*** Indicates *p* < 0.001, ** indicates *p* < 0.01, * indicates *p* < 0.05.

Furthermore, Full sample model in Table 6 includes the life satisfaction variable on the basis of Full sample model in Table 3, where the effect of physical exercise on self-rated health and mental health remains significantly positive. Compared to the results of Table 3, the coefficient is slightly reduced, indicating that life satisfaction plays a mediating role in the effect of physical exercise on self-rated health and mental health.

**Table 6:** The differentiation effect of physical exercise and life satisfaction on physical and mental health between rural and urban residents.

Variable	Full sample model		Urban model		Rural model	
	Physical health	Mental health	Physical health	Mental health	Physical health	Mental health
Physical exercise	0.022***	0.161***	0.025**	0.279**	0.019*	0.074
Life satisfaction	0.110***	1.721***	0.123***	1.967***	0.106***	1.625***
Control variable	Controlled	Controlled	Controlled	Controlled	Controlled	Controlled
Cons	3.024***	39.279***	3.215***	41.426***	2.887***	39.123***
N	9,954	9,954	2,906	2,906	7,048	7,048
Adjusted *R^2^*	0.174	0.112	0.1438	0.078	0.166	0.128

*** Indicates *p* < 0.001, ** indicates *p* < 0.01, * indicates *p* < 0.05.

To further examine the mediating effect of life satisfaction between physical exercise and physical health, the KHB method was employed. The analysis revealed that the mediating effect of life satisfaction between physical exercise and physical health was 0.004, with a 95% confidence interval of [0.002, 0.005]. The confidence intervals do not include zero, indicating that life satisfaction mediates the relationship between physical exercise and physical health, with a mediation proportion of 13.99%. The details are shown in the full sample model of the physical health model in Table 7. Therefore, hypothesis H4-1 is confirmed.

**Table 7:** The mediating effect of life satisfaction.

Category	Effect	SE	95%CI (LL)	95%CI (UL)	Effect proportion
Physical health model					
Full sample	0.004	0.001	0.002	0.005	13.99
Urban sample	0.004	0.001	0.002	0.006	13.65
Rural sample	0.003	0.001	0.002	0.005	15.17
Mental health model					
Full sample	0.055	0.010	0.037	0.075	25.64
Urban sample	0.064	0.018	0.031	0.098	18.75
Rural sample	0.053	0.011	0.032	0.077	41.91

Based on Table 7, the full sample model for mental health indicates that life satisfaction plays a significant mediating role in the relationship between physical exercise and mental health, with a mediation effect size of 0.055 and a 95% confidence interval of [0.037, 0.075]. Since the confidence interval does not include zero, this confirms that life satisfaction significantly mediates the impact of physical exercise on mental health, accounting for 25.64% of the total effect, thus supporting Hypothesis H4-2. Overall, the findings suggest that longer durations of physical exercise are associated with higher levels of life satisfaction, which in turn contribute to improved physical and mental health outcomes. Furthermore, a comparative analysis of urban and rural residents in Table 7 reveals that this mediating effect is more pronounced among rural residents. In the physical health model, life satisfaction mediates 15.17% of the effect for rural residents compared to 13.65% for urban residents. The difference is even more substantial in the mental health model, where life satisfaction accounts for 41.91% of the effect in rural areas versus only 18.75% in urban areas. These findings confirm Hypothesis H4-3 and highlight the stronger mediating role of life satisfaction in enhancing health through physical exercise among rural populations.

### Effects of physical exercise on residents’ physical and mental health: moderating effect of social class

The moderating effect of social class on the impact of physical exercise on residents’ physical and mental health is shown in Table 8 the full sample model. The interaction between physical exercise and social class has a significant effect on physical health (*b* = −0.014, *p* < 0.001) and mental health (b = −0.072, *p* < 0.05), indicating that social class moderates the relationship between physical exercise and both physical and mental health. The negative coefficients of the interaction terms indicate that the effects of physical exercise and social class on residents’ physical and mental health are mutually attenuating. Specifically, as social class rises, the impact of physical exercise on physical and mental health weakens. This suggests that physical exercise helps to narrow the gap in physical and mental health between residents of different social classes, this conclusion is consistent with the “resource substitution” theory. Therefore, hypotheses H5-1 and H5-2 are confirmed.

**Table 8:** The moderating effect of social class and the disparity between rural and urban residents.

Variable	Full sample model		Urban model		Rural model	
	Physical health	Mental health	Physical health	Mental health	Physical health	Mental health
Physical exercise	0.084***	0.488**	0.050*	0.310	0.101***	0.425*
Social class	0.063***	0.521***	0.056***	0.332*	0.065***	0.589***
Physical exercise * Social class	−0.014***	−0.072*	−0.005	−0.007	−0.019***	−0.080*
Controlled variable	Controlled	Controlled	Controlled	Controlled	Controlled	Controlled
Cons	2.960***	38.941***	3.175***	41.376***	2.823***	38.850
N	9,954	9,954	2,906	2,906	7,048	7,048
Adjusted *R^2^*	0.175	0.112	0.139	0.077	0.168	0.128

*** Indicates *p* < 0.001, ** indicates *p* < 0.01, * indicates *p* < 0.05.

As shown in Table 8, a comparison between urban and rural residents reveals that the moderating effect of social class on the relationship between physical exercise and physical and mental health is stronger among rural residents than among urban residents. Additionally, the moderating effect of social class is not significant in the urban resident sample. This indicates that there are urban–rural differences in the moderating effect of social class on the relationship between physical exercise and physical and mental health. Therefore, Hypothesis H5-3 is confirmed.

**Figure 2:**
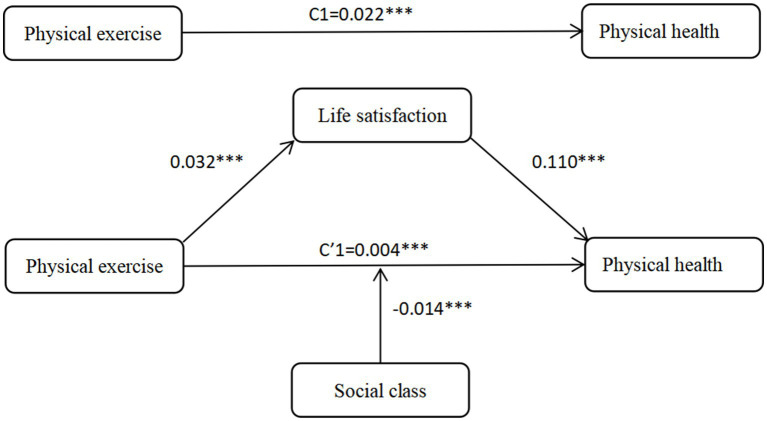
Mechanisms of physical exercise on physical health.

**Figure 3:**
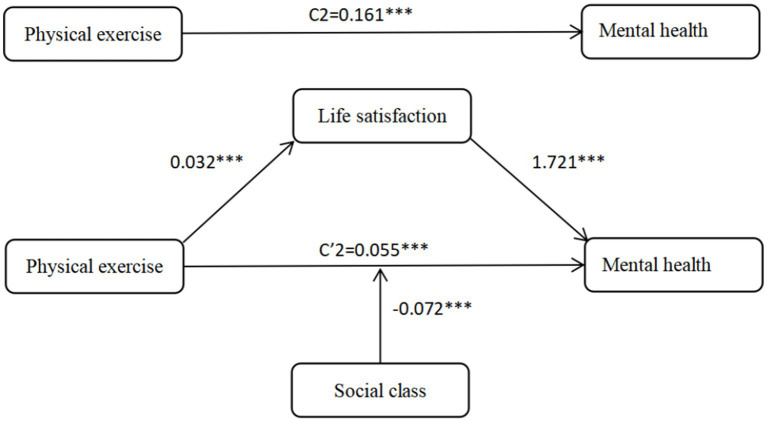
Mechanisms of physical exercise on mental health.

In order to follow the intuitive observation of the mediating effect of life satisfaction and the moderating effect of social class, we plotted Figures 2, 3 to represent the mechanism of physical activity on physical health and the mechanism of physical activity on mental health, respectively.

## Discussion

Based on research results both domestically and internationally, this study selected the CLDS 2018 data to explore the specific mechanism of physical exercise on residents’ physical and mental health. It verified the mediating role of life satisfaction and the moderating effect of social class, and further conducted an urban–rural comparison.

### The direct impact of physical exercise on residents’ physical and mental health

This study found that physical exercise significantly enhances residents’ physical and mental health. The longer the duration of physical exercise, the higher residents rate their physical and mental health. This conclusion aligns with previous research findings (Warburton and Bredin, 2018; Warburton and Bredin, 2017; Warburton and Bredin, 2016; Warburton et al., 2006a; Warburton et al., 2006b). Appropriate exercise not only improves cardiovascular function and metabolism but also helps maintain a healthy weight and body shape. Additionally, exercise releases chemicals such as endorphins and dopamine in the body, which uplift mood and thereby promote mental health.

### Mediating effects of life satisfaction

The results of this study show that life satisfaction mediates the relationship between physical exercise and both physical and mental health. Physical exercise directly affects residents’ physical and mental health, while physical exercise also affects residents’ life satisfaction, which is consistent with the results of previous studies (Reigal et al., 2014; Chekroud et al., 2018). The increase in life satisfaction can improve the physical health and mental health of residents, which is also consistent with the findings of Kim, Ziolkowski and other scholars (Proctor et al., 2009; Zullig and White, 2011). In China, physical exercise takes various forms, with residents engaging in diverse exercise types and locations. For most residents, exercise is a hobby or leisure activity that helps relieve stress, alleviate fatigue, and uplift mood through sweating. Furthermore, physical exercise promotes work-life balance and enhances quality of life, thereby boosting life satisfaction. Longer durations of exercise mean residents have more leisure time to pursue hobbies and interests, and this facilitates access to health-related information, services, and facilities, contributing to maintaining and improving residents’ physical and mental health. In short, this study introduced the mediating variable of life satisfaction, verified the mediating variable of life satisfaction between physical exercise and physical and mental health, explored the mechanism of the influence of physical exercise on physical and mental health, and enriched the research on physical and mental health.

### The moderating effect of social class

This study found that social class moderates the impact of physical exercise on residents’ physical and mental health. Analysis reveals that as residents’ social class ascends, the effect of physical exercise on their health shows a decreasing trend, indicating that the effects of physical exercise and social class on residents’ physical and mental health are mutually diminishing. On the one hand, because physical activities have strong social attributes strong social attributes, residents’ participation in physical exercise is influenced by subjective social class identification (Liu and Liu, 2021). Concurrently, engagement in physical exercise can enhance residents’ subjective class identification (Yang et al., 2022). In other words, residents from lower social classes can improve their social class identity through physical exercise, thereby promoting their physical and mental health and reducing disparities in health outcomes across different social strata. on the other hand, the resource substitution theory can be used to explain this. Individuals in higher social classes do not experience an amplification effect between the advantages of physical exercise and their socio-economic status. However, residents in lower social classes, with fewer resources, are more reliant on the physical resources available to them, leading to a resource substitution effect. Therefore, in the relationship between physical exercise and physical/mental health, the main theory at play is the resource substitution theory.

### Urban–rural comparative analysis

A comparison between urban and rural areas reveals that the impact of physical exercise is greater on urban residents than on rural residents, which is consistent with the results of previous studies (Liang and Jia, 2022; Fangfang et al., 2021). However, the mediating effect of life satisfaction on the relationship between physical exercise and health is stronger among rural residents than among urban residents, and the moderating effect of social class on the health outcomes of physical exercise is also more pronounced among rural residents. For rural residents, health is more significantly influenced by life satisfaction than by physical exercise specifically, physical exercise has no significant impact on the mental health of rural residents, while life satisfaction significantly affects both physical and mental health. Rural residents primarily improve their health through increased life satisfaction resulting from physical exercise. This finding suggests that health, particularly mental health, in rural areas is more susceptible to the influence of life satisfaction. On the other hand, this may be due to the limited availability of public sports resources in rural areas, as well as differences in exercise habits and knowledge between urban and rural residents, which prevent rural residents from achieving the same health-promoting effects from physical exercise as urban residents. Nevertheless, it is encouraging to note that physical exercise helps rural residents narrow the health gap between different social classes. This conclusion further validates the explanatory power of the “resource substitution” theory in the relationship between physical exercise and health.

The above research conclusions highlight the importance of physical exercise for physical and mental health. Therefore, the following suggestions are proposed: First, the “Healthy China” policy should continue to be promoted, with a strong emphasis on developing sports and promoting nationwide fitness. Second, the construction of sports infrastructure in communities, especially in rural areas, should be improved to provide richer facilities for residents’ daily physical exercise. By offering a variety of activities and competitions, residents should be encouraged to participate in physical exercise, thereby increasing their life satisfaction and enhancing their physical and mental health levels. Third, based on the conclusions of the resource substitution theory, particular attention should be given to increasing public sports facilities in rural areas, especially facilities that are practical, usable, and convenient for villagers. At the same time, there should be an active promotion of sports and health knowledge in rural areas, helping rural residents improve their understanding of sports-related health, which in turn will enable them to better engage in physical exercise and improve their physical and mental well-being.

### Research shortcomings and prospects

This study confirms the relationship between physical exercise, life satisfaction and physical and mental health, and explores the specific mechanisms of influence. Social class was found to play a moderating role in the relationship between physical exercise and physical and mental health. However, this study has some limitations. First, the paper discusses the frequency and duration of exercise but does not address the cumulative effect of long-term exercise on health. Then, with respect to data, the study used cross-sectional data, which cannot reveal causal relationships. Furthermore, all variables are derived from the same database, which may lead to homogeneity bias. In future research, it would be beneficial to consider using longitudinal data from different sources to enhance the credibility of the study. In addition, the possible bias in the measurement of variables, such as self-assessed health and social class, which are subjective variables and can be affected by individual subjective factors, will affect the results of the study, and the inclusion of relevant objective indicators, such as objective economic status, can be considered for comparative studies in future research.

## Conclusion

This study reveals a new mechanism by which physical exercise affects residents’ physical and mental health through life satisfaction and further explores the moderating role of social class. We found a significant correlation between physical exercise and residents’ physical and mental health levels; the longer the exercise duration, the higher the levels of residents’ physical and mental health. Additionally, physical exercise promotes residents’ physical and mental health by enhancing life satisfaction. Furthermore, we observed that social class negatively moderates the impact of physical exercise on residents’ physical and mental health, meaning that the impact of physical exercise is greater among lower social class compared to higher social class. A comparison between urban and rural residents reveals that the impact of physical exercise is greater on urban residents than on rural residents. However, the mediating effect of life satisfaction on the relationship between physical exercise and health is stronger among rural residents than among urban residents. Additionally, the moderating effect of social class on the health outcomes of physical exercise is more pronounced among rural residents compared to urban residents.

## Data Availability

The original contributions presented in the study are included in the article/supplementary material, further inquiries can be directed to the corresponding author/s.
